# Periprosthetic knee infection in octogenarians: a single institution experience at midterm outcome

**DOI:** 10.1007/s00402-023-04796-z

**Published:** 2023-02-07

**Authors:** Daniel Karczewski, Lukas Schönnagel, Christian Hipfl, Doruk Akgün, Sebastian Hardt

**Affiliations:** 1 Department of Orthopaedic Surgery, Musculoskeletal Oncology Service, Massachusetts General Hospital-Harvard Medical School, 55 Fruit Street, Boston, MA 02114 USA; 2 Department of Orthopaedic Surgery and Traumatology, Charité Berlin, University Hospital, Chariteplatz 1, 10117 Berlin, Germany

**Keywords:** Infection, Total knee replacement, Knee revision, Elderly patients, High age

## Abstract

**Purpose:**

Periprosthetic joint infections (PJI) of total knee arthroplasties (TKAs) in the elderly is among the clinically most challenging scenarios given multimorbidity combined with poor bone and soft tissue quality. Despite increasing prevalence, limited is known on PJI among this unique group of patients. As such, this study analyzed PJI characteristics, implant survivorship and non-surgical complications of octogenarians revised PJI for the knee.

**Methods:**

We identified 31 patients that were revised for PJIs of the knee between 2010 and 2019 using a single university-based registry. Mean age was 83 years (range 80–87), 48% were females, and mean BMI was 29 kg/m^2^. Mean age adjusted Charlson Comorbidity Index was 7, and mean ASA score was 3. Major causative pathogens included Coagulase-negative Staphylococci (26%), Staphylococcus aureus (13%), and Streptococci (13%). Two-stage exchange was performed in 30 patients, permanent resection arthroplasty in one joint. Kaplan–Meier survivorship analyses were performed. Mean follow-up was 4 years.

**Results:**

The 2-year survivorship free of any recurrent PJI was 96%, and there was one PJI relapse noted at 6 months. Moreover, there were three additional revisions for aseptic loosening, and one further revision for fracture. As such, the 2-year survivorship free of any revision was 87%. In addition to the aforementioned revisions, there was one additional plate osteosynthesis for a Vancouver C fracture, resulting in a 79% survivorship free of any reoperation at 2 years. Mean perioperative complication score according to the Clavien–Dindo classification was 2 out of 5. A total of three patients died: one patient 40 days after resection arthroplasty, two others 4 months and 8 months after reimplantation.

**Conclusions:**

Octogenarians revised for PJI of the knee are at low risk of recurrent infection and overall revision at 2 years. However, moderate rates of perioperative complications and mortality at short term must acknowledge before deciding upon procedure.

**Level of evidence:**

Therapeutic level IV.

## Background

The “grey wave” of an aging society with significant multimorbidity and limitations in medical resources will challenge western healthcare systems in the upcoming decades [1–3]. While total knee arthroplasty (TKA) remains among the most common procedures performed worldwide, limited is known on outcomes in patients of high age [4, 5]. Moreover, there are only a few studies to analyze complications among this unique cohort of patients, and nearly nothing is known on periprosthetic joint infections (PJI) in octogenarians [6, 7], despite an incidence of up to 1% each year [8].

Given significant gaps on knowledge in joint infections of the elderly, increasing prevalence of PJIs [8], as well as aging societies worldwide [1, 3], this article aimed to analyze octogenarians revised for PJIs of the knee. In addition to baseline and infection characteristics, we sought to determine implant survivorship, perioperative complications, and radiographic outcomes in a single university-based institution at midterm outcome.

## Patients and methods

After obtaining institutional ethics board approval, we identified 31 octogenarians treated for PJI of 31 TKAs between 2010 and 2019. Patient inclusion was performed consecutively through a standardized registry that was limited to the two-stage exchange. Mean age of the cohort was 83 years (range 80–87), mean BMI was 29 kg/m^2^ (range 19–47 kg/m^2^), and 48% were females. Mean age adjusted Charlson Comorbidity Index (CCI) [9, 10] was 7 (range 4–13), and mean American Society of Anaesthesiologists (ASA) [11] score 3 (range 2–4). The most common secondary diseases included hypertonia (74%), atrial fibrillation, diabetes, and congestive heart failure (each 26%; Table 1).

**Table 1:** Secondary diseases in patients revised for PJI of the knee

Secondary disease	Patients [no. (%)]
Hypertonia	23 (74)
Atrial fibrillation	8 (26)
Diabetes	8 (26)
Congestive heart failure	8 (26)
Post myocardial infarction	7 (23)
Peripheral arterial occlusive disease	7 (23)
Chronic pulmonary disease	7 (23)
Hypothyroidism	5 (16)
Post stroke	3 (10)
Rheumatoid arthritis	2 (6)
Tumor	1 (3)

Using the 2021 European Bone and Joint Infection Society (EBJIS) [12] criteria, 27 cases fulfilled the definition of a confirmed PJI, with the remaining 4 being considered likely PJIs. Moreover, all 31 cases were considered a confirmed PJI in consensus by one board certified orthopedic surgeon and one infectiologist. Chronic infections longer than 4 weeks occurred in 26 patients (84%), acute PJIs in the remaining 5 patients (16%), of whom three were acute hematogenous and 2 acute postoperative PJIs (6%). The McPherson classification was used to determine susceptibility to PJI [13]: systemic host grade B was present in 22 cases (71%), grade C in 9 cases (29%), local extremity grade 1 in 6 limbs (20%), grade 2 in 15 limbs (48%), and grade 3 in 10 cases (32%). Twenty-two joints were revised prior to the current intervention (mean 2, range 1–8), 13 of whom for PJI (42%). Leading pathogens included Coagulase-negative *Staphylococci* (26%), *Staphylococcus aureus* (13%), and *Streptococci* (13%) (Table 2).

**Table 2:** Infection characteristics of knees revised for PJI

Pathogens (identified in at least two samples)	Knee PJI [no. (%)]
Staphylococcus	12 (39)
*Coagulase-negative Staphylococcus (CNS)*	8 (26)
*Staphylococcus aureus*	4 (13)
Streptococcus species	4 (13)
Candida	2 (6)
E. coli	2 (6)
Other microorganisms	7 (23)
Culture-negative	9 (29)
Polymicrobial cases	7 (23)

Two-stage exchange was performed in 30 joints, whereas one patient died before undergoing subsequent reimplantation. All patients received a non-articulating cement spacer containing two or more high dose antibiotics of up to 4 g per 40 g cement. In case of susceptible pathogens and culture-negative conditions these included 1 g gentamicin and 1 g clindamycin with an additional 2 g of vancomycin per 40 g cement [14]. All 5 cases of acute PJI were treated with a two-stage exchange instead of an attempted DAIR given well established contraindications to the latter. Poor soft tissue and bone quality was noted in 2 patients with multiple prior revisions to the joint [14]. One additional patient had poor soft tissue quality through diabetes and morbid obesity (BMI 45 kg/m^2^) [15]. The fourth patient had advanced stage peripheral arterial occlusive disease (Fontaine grade III–IV) [16], and the fifth involvement of a difficult to treat pathogen (*Enterococcus faecalis*) [17]. No patient required a rotation flap.

Empirical antimicrobial therapy consisted of ampicillin/sulbactam (3 × 3 g, i.v.) [14]. In case of multiple prior revisions, bacteremia, known methicillin-resistant Staphylococcus aureus status, and suspected low-grade PJIs the regimen was moreover augmented with vancomycin (2 × 1 g, i.v.) [18]. Following pathogen cultivation, the regimen was tailored according to the antibiotic susceptibility, based on the recommendations of Zimmerli et al. [19] and in accordance with our infection disease specialists. Antibiotics were continuously administered according to a standardized regimen for a total of 12 weeks [18]. Non-biofilm active i.v. antibiotics were given for the first 2 weeks after resection arthroplasty, followed by oral antibiotics without rifampin for an additional 4 weeks. After reimplantation, i.v. antibiotics without antibiofilm activity were administered for one week, and the 12-week course completed by a 5-week course of oral biofilm active antibiotics.

If CRP was increased or other signs of infection were present antibiotic therapy was prolonged. In return, patients without prior revisions and good soft tissue conditions were possible candidates for a short-term interim of as few as 4 weeks [14, 19]. As such, mean time between resection arthroplasty and reimplantation was 66 days (range 33–241), with three patients undergoing irrigation and debridement for wound healing delay following resection arthroplasty (10%). Mean length of antibiotic therapy between resection and reimplantation was 9 weeks (excluding cases of interim debridement). Mean length of antibiotic therapy after reimplantation was 8 weeks (excluding cases of reinfection). Three patients received oral long-term antibiotic therapy of more than 6 months.

Mean length of hospital stay was 20 days (range 6–67 days) and 16 days (range 7–67 days) at resection and reimplantation, respectively. Within 2 years, 3 patients were revised, 3 died, and 11 truly had a follow-up of less than 2 years. Among patients alive and unrevised, mean follow-up was 4 years (range 2–6 years). Mean follow-up among all 31 patients was 2.5 years, and all 31 patients used for analysis.

Study outcomes were defined as survivorship free of any PJI, any infection, any revision and any reoperation. Recurrence in PJI was defined according to the 2021 EBJIS criteria [12], and any infection considered any PJI and additional supra fascial wound infection. A revision was considered any component removal and was per definition a reoperation, whereas any surgery with complete implant retention was per nature a reoperation, but not a revision. The Clavien–Dindo classification [20] was used to evaluate perioperative complications. Radiographic analysis was performed according to the standardized Knee Society radiographic evaluation form [21]. Results were reported as means with ranges, and survivorship analysis based on Kaplan–Meier curves [22]. Calculations were performed using SAS version 9.4 (SAS Institute, Inc., Cary, North Carolina).

## Results

The 2-year survivorship free of any recurrent PJI was 96%. There was one recurrent PJI at 6 months treated with resection arthroplasty. Moreover, one additional surgical site infection was noted at 10 days, resulting in a 92% survivorship free of any infection at 2 years. There were an additional 4 revisions: one for a periprosthetic fracture at 6 days, two for aseptic loosening at 6 months and 3 years, and one for dislocation with subsequent change of the acetabular component at 3 years. As such, the 2-year survivorship free of any revision was 87%. Finally, there was one additional plate osteosynthesis for a Vancouver C fracture at one month, resulting in a survivorship free of any reoperation of 79% at 2 years.

Mean Clavien–Dindo score were 2 out of 5 at both time of resection and reimplantation (range in both, 0–5). Likewise, anemia and acute kidney injury were the most common complications occurring in patients at both resection and reimplantation during their stay at the orthopedic ward (Table 3). None of the acute kidney injuries occurred through antibiotics, and no patient needed to switch antibiotics due to side effects or risk of end organ damage. Forty three percent of patients were transferred to the ICU after reimplantation (mean 3 days; range, 1–19 days), with 40% being later on released to their home (*n* = 12), 57% to a geriatric department (*n* = 17), and one remaining in the hospital care until dying at 4 months. Outside of the reoperations outlined earlier no patient was readmitted to our ward after final release.

**Table 3:** Perioperative complications at resection and reimplantation

Complication	Resection [no. (%)]	Reimplantation [no. (%)]
Anemia	26 (84)	24 (77)
Acute kidney injury	4 (13)	4 (13)
Delirium	4 (13)	2 (7)
Lung arterial emboly	1 (3)	1 (3)
Pneumonia	1 (3)	1 (3)
Myocardial infarction	1 (3)	0
Death	1 (3)	0

Among the unrevised and successfully reimplanted 30 TKAs, 12 had a radiographic follow-up of one month or more (mean follow-up 2 years, range 49 days–6 years). Lucency without signs of definite loosening were identified in three cases, located at the medial tibial tray at 4 months, distal tibia stem at 1 year, and femoral and tibial components at 4 years of follow-up, respectively. Moreover, definite loosening at the ventral tibial component was noted at 8 months in a fourth case with the patient refusing a further revision.

## Discussion

Both primary TKAs and PJIs will increase significantly over the next decades [8]. This study is the first to analyze revisions for PJI of the knee in octogenarians at midterm follow-up. We found such patients to have a low rate of recurrent PJI (4%) with moderate rate of reoperations (21%) at 2 years. Moreover, patients were at high risk of non-surgical complications, although severe complications, including acute kidney injury (13%) and perioperative mortality (3%) remained low.

Limited is known on octogenarians revised for PJI of the knee [6, 7]. We were able to show that such patients represent a highly challenging cohort with significant comorbidities. In fact, around one in four patients had either atrial fibrillation, congestive heart failure, or a prior myocardial infarction at time of revision. Moreover, one in four patients was diagnosed with diabetes or peripheral arterial occlusive disease, known to directly impact success of revision [23, 24]. These profound risk factors are also indirectly reflected in a poor McPherson limb grade and high rate of previous revisions. Importantly, these factors combined with poor bone and soft tissue quality had direct impact on clinical procedures, as all five cases of acute onset PJI were treated with a two-stage exchange instead of an attempted implant retention [25].

Knowledge on surgical outcomes following revisions for PJI of the knee in the elderly is limited to a few case series, and to the best of our knowledge, the current report is the first midterm study in octogenarians to this date. We found the 2-year reinfection rate to be 8%, and as such lower than previously reported for the two-stage exchange in the general PJI population [26, 27]. Of note, all recurrent infections occurred in the first year, similar to one previous study on short-term outcomes among patients older than 79 years revised for PJI [28].

A possible alternative to the two-stage exchange might be the usage of a permanent spacer or the one-stage exchange, with the latter being limited to cases with well retained bone stock and good soft tissue conditions [29, 30]. Importantly, both strategies offer the advantage of reduced total time spent in the hospital, possibly presenting a feasible option for patients with limited life expectancy and those unwilling to undergo a staged procedure. In addition, less time spent in the hospital might reduce overall complications, while simultaneously increasing overall cost efficiency [31]. On the other hand, however, the two-stage exchange might allow for a better short- and midterm functionality with possibly increased bone protection as opposed to a permanent spacer [29, 32].

All but one patient in our study experienced at least one perioperative complication. Importantly, around 10% were of severe to potentially life-threatening nature, including lung arterial emboly, pneumonia, and myocardial infarction. Consequently, we found 40% of patients to require a postoperative ICU stay, with most patients in need of a geriatric rehabilitation thereafter. Despite that, only one patient died in the extended perioperative period (3%). Of note, this finding contradicts one report on octogenarians admitted to the ICU for PJI (28% mortality rate) [33], and rather reflects mortality rates of the general PJI population [34, 35]. We believe this discrepancy to be caused by a selection bias given a specialized university-based department, as well as a potential survival bias, as most cases were of chronic rather than acute presentation.

This study must be viewed in the context of its limitations. Foremost, we report of a single center retrospective cohort, and despite including one of the largest series to date, of a comparably low number of cases. In addition, the follow-up was short-term only. Of note, only 12 patients had a short-term radiographic follow-up, limiting knowledge on the state of the TKA. Moreover, treatment occurred in a highly specialized university center by an interdisciplinary team possibly not reflecting the general treatment available. Finally, patient reported outcomes and functionality were not accessed, reducing comparability with other approaches such as chronic antibiotic suppression therapy [36].

To give a resume, this is the first article to analyze octogenarians revised for PJIs of the knee at short to midterm follow-up. We found less than one in ten patients to experience a recurrent PJI within 2 years. While the majority of patients experienced moderate non-surgical complications, both severe complications, as well as mortality remained low during the perioperative phase. Further studies focusing on mid- and long-term follow-up will be necessary.

## Data Availability

Made available upon request.
